# A qualitative evaluation of priority-setting by the Health Benefits Package Advisory Panel in Kenya

**DOI:** 10.1093/heapol/czac099

**Published:** 2022-11-14

**Authors:** Rahab Mbau, Kathryn Oliver, Anna Vassall, Lucy Gilson, Edwine Barasa

**Affiliations:** Department of Global Health and Development, London School of Hygiene and Tropical Medicine, Keppel Street, London WC1E 7HT, UK; Health Economics Research Unit, KEMRI Wellcome Trust Research Programme, P.O. BOX 43640-00100, 197 Lenana Place, Nairobi Kenya; Department of Public Health Environment and Society, London School of Hygiene and Tropical Medicine, Keppel Street, London WC1E 7HT, UK; Department of Global Health and Development, London School of Hygiene and Tropical Medicine, Keppel Street, London WC1E 7HT, UK; Department of Global Health and Development, London School of Hygiene and Tropical Medicine, Keppel Street, London WC1E 7HT, UK; Health Policy and Systems Division, School of Public Health and Family Medicine, University of Cape Town, Anzio Road, Observatory 7925, South Africa; Health Economics Research Unit, KEMRI Wellcome Trust Research Programme, P.O. BOX 43640-00100, 197 Lenana Place, Nairobi Kenya; Centre for Tropical Medicine and Global Health, Nuffield Department of Medicine, University of Oxford, Old Campus, Roosevelt Drive, Oxford OX3 7LG, UK; Institute of Healthcare Management, Strathmore University, Karen Ole Sangale Road, P.O. BOX 59857-00200, Nairobi, Kenya

**Keywords:** Qualitative evaluation, macro (national) level, healthcare priority-setting process, Health Benefits Package Advisory Panel, Kenya

## Abstract

Kenya’s Ministry of Health established the Health Benefits Package Advisory Panel (HBPAP) in 2018 to develop a benefits package for universal health coverage. This study evaluated HBPAP’s process for developing the benefits package against the normative procedural (acceptable way of doing things) and outcome (acceptable consequences) conditions of an ideal healthcare priority-setting process as outlined in the study’s conceptual framework. We conducted a qualitative case study using in-depth interviews with national-level respondents (*n* = 20) and document reviews. Data were analysed using a thematic approach. HBPAP’s process partially fulfilled the procedural and outcome conditions of the study’s evaluative framework. Concerning the procedural conditions, transparency and publicity were partially met and were limited by the lack of publication of HBPAP’s report. While HBPAP used explicit and evidence-based priority-setting criteria, challenges included lack of primary data and local cost-effectiveness threshold, weak health information systems, short timelines and political interference. While a wide range of stakeholders were engaged, this was limited by short timelines and inadequate financial resources. Empowerment of non-HBPAP members was limited by their inadequate technical knowledge and experience in priority-setting. Finally, appeals and revisions were limited by short timelines and lack of implementation of the proposed benefits package. Concerning the outcome conditions, stakeholder understanding was limited by the technical nature of the process and short timelines, while stakeholder acceptance and satisfaction were limited by lack of transparency. HBPAP’s benefits package was not implemented due to stakeholder interests and opposition. Priority-setting processes for benefits package development in Kenya could be improved by publicizing the outcome of the process, allocating adequate time and financial resources, strengthening health information systems, generating local evidence and enhancing stakeholder awareness and engagement to increase their empowerment, understanding and acceptance of the process. Managing politics and stakeholder interests is key in enhancing the success of priority-setting processes.

**Key messages**

- Evaluation of healthcare priority-setting processes highlights what happens in practice and provides opportunities for improvement where actual practice does not align with normative procedural or outcome conditions.
- HBPAP’s priority-setting process for health benefits package development partially fulfilled the normative procedural and outcome conditions due to internal and external limitations.
- Priority-setting processes for health benefits package development in Kenya could be improved by publicizing the outcome of the process, allocating adequate time and financial resources, strengthening health information systems, generating local evidence, enhancing stakeholder awareness and engagement and managing politics and stakeholder interests.

## Introduction

Healthcare priority-setting refers to the process of making decisions regarding allocation of resources among programmes, services and patient groups competing for scarce resources (Mckneally *et al.*, 1997). It occurs at all levels of the health system namely the micro (provider-patient); meso [sub-national or organizational (e.g. hospital)] and macro (national) levels (Mckneally *et al.*, 1997). Healthcare priority-setting can be done implicitly or explicitly (Chalkidou *et al.*, 2016). Implicit priority-setting processes are *ad hoc* and non-transparent, while explicit priority-setting processes are systematic, transparent, inclusive and driven by evidence, social values and deliberation among relevant stakeholders (Chalkidou *et al.*, 2016).

Health system resource constraints and wastage have led to growing demands for countries to adopt explicit priority-setting processes to inform universal health coverage (UHC) (World Health Organization, 2014; Chalkidou *et al.*, 2016). UHC means ensuring that everyone in need can obtain good-quality promotive, preventive, diagnostic, curative, palliative and rehabilitative health services without financial troubles (World Health Organization, 2010). Explicit priority-setting processes can inform UHC decisions such as development of a health benefits package—a set of defined health services for a specified population that are funded from pooled resources (Bobadilla *et al.*, 1994; Glassman *et al.*, 2017).

As countries adopt explicit priority-setting processes, there is an accompanying demand to evaluate them (Smith *et al.*, 2012). This demand is driven by the public’s interest in decision-makers demonstrating the fairness, legitimacy, accountability and transparency of healthcare priority-setting processes, given the complexity and distributive conflicts associated with allocating scarce resources across competing uses (Ham and Coulter, 2001; Martin and Singer, 2003). Evaluation refers to the systematic collection and analysis of data to determine the merit of a process, policy or program (Smith *et al.*, 2012).

Evaluation of healthcare priority-setting processes can be guided by normative conditions drawn from two philosophies—proceduralism and consequentialism (Coulter and Ham, 2000). Proceduralism judges whether a healthcare priority-setting process follows acceptable ways of doing things, while consequentialism judges whether a healthcare priority-setting process leads to acceptable outcomes (Jan, 2014). A good healthcare priority-setting process fulfils the normative procedural and/or outcome conditions. Evaluation of healthcare priority-setting processes highlights what happens in practice and provides opportunities for improvement where actual practice does not align with normative procedural or outcome conditions (Smith *et al.*, 2012).

Globally, empirical studies on evaluation of healthcare priority-setting processes remain limited (Martin and Singer, 2003; Smith *et al.*, 2012). This gap is wider for macro level processes and low- and middle-income countries (LMICs). A literature review on evaluation of priority-setting processes in the health sector conducted in 2015 identified 27 empirical studies, of which only 5 and 7 covered the macro level and LMICs, respectively (Barasa *et al.*, 2015). Given this gap, evaluating how well priority–setting processes are conducted at the macro level in LMICs is, therefore, a substantially relevant health systems research question.

In Kenya, a lower-middle-income country, studies on evaluation of healthcare priority-setting processes at the macro level remain limited. Recent publications include a multi-country study on evaluation of macro level priority-setting for COVID-19 preparedness plans (Kapiriri *et al.*, 2021) and priority-setting for non-communicable diseases (Wanjau *et al.*, 2021). Previous studies have largely been conducted at the meso level, namely hospital level (Barasa *et al.*, 2017) and county level (Bukachi *et al.*, 2014; Nyandieka *et al.*, 2015; Waithaka *et al.*, 2018b).

As part of the UHC reforms, Kenya’s Ministry of Health (MOH) established the Health Benefits Package Advisory Panel (HBPAP) in 2018 to develop a benefits package for UHC using an explicit priority-setting process. This study seeks to contribute to the literature on evaluation of macro level priority-setting processes by describing and qualitatively evaluating the extent to which the priority-setting process conducted by HBPAP fulfilled the key elements of an ideal healthcare priority-setting process.

## Methods

### Study setting

Kenya’s governance structure is devolved with 1 national government and 47 semi-independent county governments. In the health sector, the national government has policy and regulatory roles, while county governments oversee service provision (The Republic of Kenya, 2010). Kenya’s health sector is financed from three major sources namely the government, households and donors which accounted for 46%, 35.5% and 18.5% of the total health expenditure in 2019, respectively (World Health Organization, 2019).

In the public health sector, purchasing, which refers to the transfer of pooled funds to healthcare providers for health service delivery (World Health Organization, 2010), is done by the following purchasing organizations. The MOH purchases services from national referral hospitals using global budgets derived from national government revenue. The county governments purchase services from county public hospitals and primary healthcare facilities using line-item budgets derived from the county revenue fund. Finally, the National Health Insurance Fund (NHIF) purchases services from public healthcare facilities using capitation, case-based payments, fee-for-service and rebates derived from premium contributions from its members (Mbau *et al.*, 2018; 2020). The NHIF is the largest health insurer in Kenya covering ∼17.8% of the population (Ministry of Health, 2018a). In 2018, the Kenyan government identified UHC as one of its four key aspirational development agenda and planned to roll it out as a 12-month pilot in four counties in 2019 before a progressive nationwide scale-up across all counties starting from 2020.

### Study design

We used a qualitative case study approach to explore people’s perspectives and experiences of the priority-setting process given that priority-setting is a context-dependent social process (Coulter and Ham, 2000; Martin and Singer, 2003). The case in this enquiry was the priority-setting process for health benefits package development conducted by HBPAP within the MOH in Kenya.

### Conceptual framework

**Figure 1.**
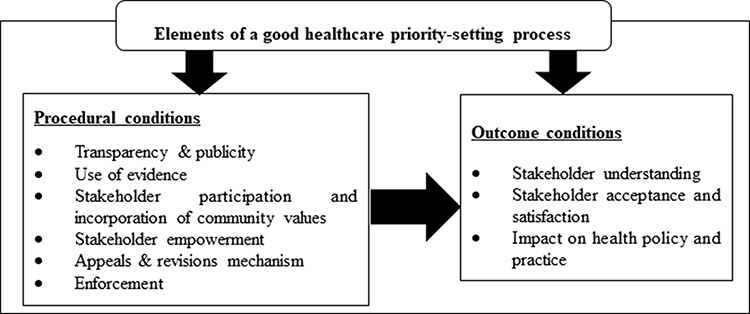
Framework for evaluating healthcare priority-setting processes, adapted from (Barasa *et al.*, 2015)

We adapted the Barasa et al.’s conceptual framework(Figure 1) to describe and qualitatively evaluate the extent to which HBPAP’s priority-setting process fulfilled the key conditions of a good priority-setting process. We chose this framework because it is based on a synthesis of empirical and conceptual literature on normative procedural and outcome conditions that stakeholders across countries of different income levels are considered essential for a good, fair, legitimate, socially justifiable or successful priority-setting process at the macro and meso levels of the health system (Barasa *et al.*, 2015). We also chose this framework because it drew on concepts from theoretical literature on deliberative democracy and procedural justice which spanned beyond the health sector (Barasa *et al.*, 2015).

According to Barasa et al., the procedural conditions of a good priority-setting process include transparency, use of evidence, stakeholder participation and incorporation of community values, stakeholder empowerment, revisions and appeals mechanism and enforcement. The outcome conditions include stakeholder understanding, stakeholder acceptance and satisfaction and impact on health policy and practice (Barasa *et al.*, 2015). The definitions of these conditions are provided in Supplementary Material. We used this framework to develop the study’s interview topic guide and to guide data analysis.

### Study population and sampling strategy

We sampled study participants through purposive and snowballing techniques. The aim was to generate a deep understanding of the experience of priority-setting through engaging information-rich participants rather than interviewing a representative sample of every stakeholder involved in the process (Sandelowski, 1995). The purposive criterion was a participant’s involvement in HBPAP’s priority-setting process as a HBPAP member. HBPAP members were then asked to nominate other stakeholders who were involved in the priority-setting process, aiding access to hard-to-reach elites. Sampling was stopped at the point of data saturation. A total of 20 stakeholders (Table 1) were interviewed. None of the participants’ demographic information is provided to maintain confidentiality and anonymity.

**Table 1.** List of participants

Category	Subcategory	Number
HBPAP participants	HBPAP members	*n* = 9
Non-HBPAP participants	MOH	*n* = 4
	Academic and research organizations	*n* = 2
	Public health sector agencies	*n* = 2
	Development partners	*n* = 3
Total		*n* = 20

### Data collection methods

We collected data through in‐depth interviews, document reviews and field notes between October and December 2021.

#### In-depth interviews

We contacted participants via telephone and email to request their participation; none declined. Before the interview, each participant reviewed the research information sheet and gave informed consent. The interviews were conducted via face–face in the participant’s office or via Zoom videoconferencing. Each interview was conducted in English, audio-recorded with an encrypted recorder and lasted approximately 1 h. For the interviews, we used a semi-structured topic guide that was informed by the key elements of the study’s conceptual framework.

#### Document reviews

We identified documents with potentially relevant information on HBPAP’s priority-setting process (Table 2) from interview participants and online media platforms.

**Table 2.** List of reviewed documents

Types of documents	Examples (all available in electronic versions)
Government documents	Gazette notice Vol. CXX- No.69 Kenya Health Policy 2014–2030 Draft UHC report Policy brief on the harmonized benefits package Online media reports on MOH web portals
HBPAP documents	Procedural documents—attendance sheets and memos HBPAP reports and annexes HBPAP presentations
Semi-autonomous government agencies documents	NHIF reforms panel report

#### Field notes

We took handwritten field notes during interviews to record audio information when participants requested for the tape recorder to be switched off. We also used field notes as aids for critical reflection of emerging themes and refinement of the topic guide. After the interviews, we transferred the field notes to Microsoft word documents to prevent loss of data through forgetfulness. Each field note was linked to the respective interview through dates and numbers.

### Data analysis

All audio files were transcribed verbatim by a transcription agency. We reviewed all transcripts for transcription accuracy by comparing them to the audio files and cleaning them where necessary. All transcripts, field notes and electronic documents were uploaded to NVIVO Pro software QSR International, Burlington, Massachussetts for effective organization during data analysis. We used the Braun and Clarke six-step approach to analyse the data thematically (Braun and Clarke, 2006).

In Step 1, we familiarized ourselves with the data through immersion. In Step 2, we generated a list of codes related to the elements of the study’s conceptual framework. In Step 3, we developed themes by identifying patterns between codes and grouping similar codes together. In Step 4, we checked for coherence between the list of themes and the coded data extracts. In Step 5, we applied the approved themes across the data. In Step 6, we produced a synthesis of the findings related to the evaluation of HBPAP’s priority-setting process and linked these findings to broader empirical literature. These findings were reviewed and approved by all authors.

### Trustworthiness

We built trustworthiness in the study findings by using different methods of data collection (method triangulation), interviewing multiple participants to identify multiple perspectives (data source triangulation), iterative questioning through rephrasing of questions and use of probes and holding peer debriefing sessions with the study team.

### Reflexivity

All authors have participated in priority-setting processes across different LMICs. Specifically, two authors have participated in previous priority-setting processes in Kenya which influenced their interest in the study topic and methodology including sampling of participants, data collection methods and data analysis.

## Findings

### Description of HBPAP’s priority-setting process

HBPAP was appointed by the Cabinet Secretary for Health (equivalent to MOH) on 8 June 2018. It consisted of HBPAP members who were experts with different technical backgrounds and experience in priority-setting such as health financing and health systems experts, epidemiologists, clinicians, actuaries and county government representatives. HBPAP was formed to, among others (Table 3), develop a benefits package for UHC using an explicit priority-setting process within 60 days (The Kenya Gazette, 2018). This UHC health benefits package would be funded by the Government of Kenya and purchased through the NHIF (Ministry of Health, 2018a). The health benefits package would be piloted in four counties before being implemented nationally across all 47 counties.

**Table 3.** HBPAP’s roles and responsibilities as assigned by the MOH (The Kenya Gazette, 2018)

(1) Develop criteria for assessing and appraising health technologies. (2) Develop an evidence-based health benefits package for Kenyans. (3) Propose provider payment methods and rates for the health benefits package. (4) Define a framework for institutionalizing health technology assessment in Kenya.

To develop the benefits package, HBPAP identified 10 priority-setting criteria using a nominal group technique—a structured approach that involves collective deliberation and consensus. Table 4 outlines these criteria, their definition and how they were operationalized.

**Table 4.** The 10 priority-setting criteria for assessing and appraising services (Ministry of Health, 2018b)

Criteria	Definition	Data sources and operationalization
Effectiveness and safety	The service improves health status and is safe for use	Clinical guidelines and pathways
Burden of disease	The service addresses disease conditions that affect many Kenyans	Nationally representative surveys, burden of disease data from the Institute for Health Metrics Evaluation, routine data from Health Management Information Systems
Severity of disease	The service addresses the most debilitating illnesses in Kenya	Disability weights from burden of disease studies
Catastrophic health expenditure	Coverage of the service reduces the risk of poverty associated with an individual’s access to that service	Nationally representative surveys and analyses
Cost-effectiveness	The service offers the best possible use of available resources to improve health status	Cost-effectiveness databases—Tuft Cost-Effectiveness Analysis, Disease Control Priorities 3 and World Health Organization—Choosing Interventions that are Cost-Effective
Affordability	Kenya has the financial resources to cover the costs associated with the provision of the service.	Budget impact analysis using expenditure and cost data from secondary sources
Feasibility: health workforce requirements	Kenya has the human resource capacity required to provide the service	Kenya health workforce report data and information from professional regulatory bodies
Feasibility: Service and health products and medical technology requirements	Kenya has other health technologies required to support provision of the service	Service readiness surveys
Equity	Provision of the service addresses disparities in access and utilization of needed health services	Benefit incidence analysis of services
Congruence with existing priorities	The service aligns with the priorities identified in the constitution and health sector policies	Document reviews of MOH policies

**Figure 2.**
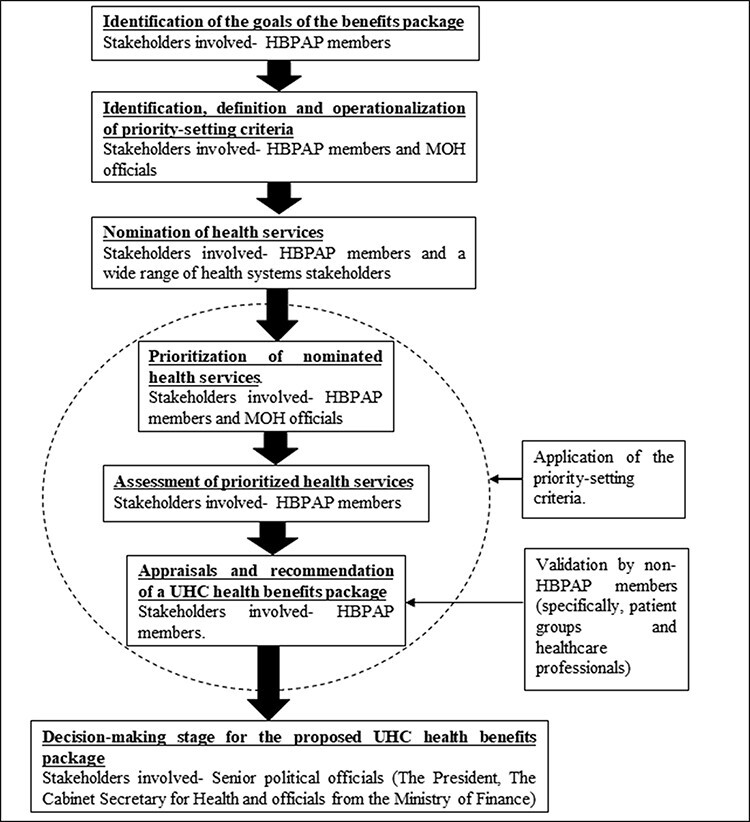
HBPAP’s healthcare priority-setting process for health benefits package development. Source: authors, based on participants’ reports and document reviews

Next, HBPAP started with a long list of health services that was drawn from existing benefit packages in the health sector, namely the Kenya Essential Package for Health that was offered in public healthcare facilities and the NHIF’s general scheme benefits package. HBPAP then invited nominations for additional services from a wide range of health systems stakeholders. HBPAP prioritized the list and conducted assessments and appraisal of services using the 10 priority-setting criteria. Assessments relied on published literature and secondary data because of short timelines and limited primary data. Services that met the priority-setting criteria following appraisal were included in the benefits package that was submitted as a proposal to the MOH for final decision-making. Figure 2 outlines HBPAP’s priority-setting process.

### Evaluation of HBPAP’s priority-setting process

Overall, HBPAP’s priority-setting process partially fulfilled the normative procedural and outcome conditions specified by the study’s conceptual framework. This partial fulfilment was due to several limitations as described further below.

### Procedural conditions

#### Fairly transparent but less publicly available information on HBPAP’s priority-setting process

The composition, roles and responsibilities of HBPAP were transparent and publicly available through a Government Gazette Notice (The Kenya Gazette, 2018). According to the Notice, HBPAP was constituted as an advisory body for the MOH. It consisted of 1 chairman, 14 members and 2 joint secretaries. The roles and responsibilities of HBPAP and non-HBPAP members in the priority-setting process (Table 5) were transparent to those involved. These were explicitly outlined in HBPAP’s Internal Procedures Manual (Ministry of Health, 2018b). However, the Manual was only accessible to HBPAP members and MOH officials which undermined its public availability.

**Table 5.** Roles and responsibilities of HBPAP and non-HBPAP members (Ministry of Health, 2018b)

Category of stakeholders	Roles and responsibilities in the priority-setting process
HBPAP members	Identify, define and operationalize the priority-setting criteria Assess and appraise nominated services Propose a health benefits package
Non-HBPAP members	Nominate services Advise on the priority-setting criteria and proposed health benefits package
Senior political leadership (The President, the Cabinet Secretary for Health and senior officials from the Ministry of Finance)	Advise on the proposed benefits package Make the final decision on the implementation of the proposed health benefits package

HBPAP’s priority-setting process was transparent. Transparency was achieved through generation and use of priority-setting criteria to inform selection of services for the health benefits package and involvement of stakeholders in the different steps of the priority-setting process. Transparency was also achieved through development of a detailed report on the process which made each step of the priority-setting process clear, replicable and auditable.

*‘The panel ensured that every stage of the process was open in compliance with the constitutional dispensation that gives Kenyans a right to access and demand information. The panel also wanted to win the confidence of all stakeholders so that the ownership of the document was acquired ab initio until the end’* HBPAP participant 4

While HBPAP’s priority-setting process was transparent, publicity around it was limited by the failure to publish HBPAP’s report which had outlined explicitly the methodology used to design the health benefits package.

*‘The report was to enable the government to decide on what to roll out as a benefits package for UHC and what to communicate to the public. However, the government did not adopt the report, so it has never been made public’* Development partner, participant 2

There was also lack of transparency and publicity of the final decision-making process in which the senior political leadership consisting of the President, the Cabinet Secretary for Health and officials from the Ministry of Finance made the final decision on the implementation of HBPAP’s proposed health benefits package.

*‘There was no transparency in the final decision-making about whether or not to implement the benefits package that we had developed. We, therefore, do not know how the decision was made and what factors were considered’* HBPAP participant 1

#### Adequate identification but limited application of priority-setting criteria

HBPAP identified commonly used priority-setting criteria (Table 4) from peer-reviewed literature, organizational websites and national health policy documents (Ministry of Health, 2018b). However, the application of the criteria was undermined by limited quality of evidence (missing and/or outdated evidence) on the criteria due to inadequate availability of good-quality local primary or published data and inadequate health information systems. The application of the cost-effectiveness criterion was undermined by the lack of local cost-effectiveness threshold. Furthermore, the 60-day timeline was considered too short to allow application of the criteria across all nominated services. Finally, political interference overruled technical evidence in the final decision-making phase.

‘*We had a time crunch. We only had 60 days…We could not intensively subject every service to the 10 criteria’* HBPAP participant 7

*‘The criteria were very important to us from a technical and political perspective because this was a political and technical process. We had to tell technocrats and politicians why we were including A and not B and the criteria were majorly our point of argument but somehow politics took the larger pie of that cake in the final stage.’* HBPAP participant 6

#### Adequate participation of stakeholders and incorporation of community values

There was good participation by HBPAP members in the priority-setting process as shown by their good attendance and contribution during meetings. In addition, a wide range of non-HBPAP stakeholders participated in the priority-setting process. Broadly, they included MOH technocrats and bureaucrats, health professional associations and unions, public and private health sector agencies, civil society organizations, development partners and middle and senior political leaders from the four pilot counties and the national government. Stakeholder participation was through stakeholder engagement forums that were financially and administratively supported by development partners and semi-autonomous government agencies. Stakeholder participation was important for incorporating stakeholder values and preferences and fulfilling the constitutional principles of procedural justice of public participation, right to information, accountability and transparency (Ministry of Health, 2018b).

*‘The panel consulted different stakeholders in their process of work so that it was not just their views but the views of the whole sector’* MOH participant 1

While HBPAP had tried to be as inclusive as possible, participants reported that some stakeholders, such as informal sector workers and international non-governmental organizations supporting disease-specific programmes, had been left out of the priority-setting process. They also reported that MOH bureaucrats should have been assigned more roles in the process to increase their level of ownership and acceptance of HBPAP’s priority-setting process and proposed health benefits package.

*‘The Heads of Departments within the MOH were not extensively engaged in the process…That was a major pitfall because ownership by the MOH was not there. They just looked at the report as HBPAP’s report. They didn’t seem to accept it’* HBPAP participant 5

According to participants and the documents reviewed, extensive stakeholder participation and consideration of other shared community values were limited by the lack of a budget for HBPAP’s operational costs and the 60-day timeline.

*‘They tried; they met with different groups, but they had 60 days. What can you do in 60 days? 60 days was a very short time’* Development partner, participant 3

#### Adequate empowerment of HBPAP members but limited empowerment of non-HBPAP members

HBPAP members were empowered to participate in the priority-setting process through (1) their appointment into HBPAP via a legal gazette notice, (2) HBPAP’s semi-autonomous nature that provided them with the decision space to conduct the priority-setting process, (3) their technical expertise and professional experience in priority-setting processes, (4) the positive organizational culture within HBPAP which was characterized by strong and supportive leadership, commitment and mutual respect among members and (5) political goodwill from the senior political leadership who not only appointed them but also dedicated time to engage with them.

*‘There was support from the leadership to have this completed. The former Cabinet Secretary spent hours in the boardroom with the panel discussing the benefit package. There was also support and engagement from the President which pushed the panel through’* MOH participant 2

However, HBPAP members’ empowerment was undermined by the lack of allocation of office space and financial resources which limited their operational activities such as stakeholder engagement forums. Their empowerment was also undermined by the 60-day timeline which limited the extent to which HBPAP members could meaningfully employ their technical expertise.

*‘Time was a very scarce resource. In future, it would be good to allocate more time so that the panel can work well without rushing through the process’* HBPAP participant 9

Non-HBPAP members were empowered to participate in the priority-setting process through stakeholder engagement forums. HBPAP held different engagement forums for different stakeholders to minimize dominance of certain groups of stakeholders over others. However, empowerment of non-HBPAP members was limited by the inherently technical nature of the priority-setting process. Consequently, non-HBPAP members with technical expertise and prior experience in priority-setting were more empowered to contribute and influence the process than those without.

*‘Stakeholders’ technical knowhow influenced their participation. Donors in health financing had technical capacity to participate in the process but majority did not have health systems experience hence did not fully engage with the process’* HBPAP participant 6

#### Limited appeals and revisions mechanism

Non-HBPAP members could challenge or provide feedback on the decisions made by HBPAP through stakeholder engagement forums or HBPAP’s official email account. However, this feedback process was limited by the 60-day timeline for the priority-setting process. There was also no formal mechanism to revise and appeal the final decision of the priority-setting process because the senior political leadership did not adopt the proposed health benefits package.

*‘An appeals or revision process did not occur because it was not clear what the package was. It must be clear what the package is for people to appeal or revise it. However, because the MOH decided to go another way, there was nothing to appeal’* HBPAP participant 1

#### Adequate availability and application of enforcement measures

HBPAP’s Internal Procedures Manual provided a good mechanism for ensuring that HBPAP’s priority-setting process adhered to the key principles of explicit priority-setting. The manual covered: (1) meeting and decision-making procedures including code of conduct (impartiality and objectivity); (2) communication and stakeholder engagement strategies; (3) terms of reference such as membership, key roles and responsibilities, deliverables and reporting lines within and outside of HBPAP; (4) affirmation of commitment to HBPAP’s activities and deliverables and (5) conflict-of-interest statement which outlined the types, declaration and management of conflicts of interest. Enforcement was also achieved through oversight and leadership provided by the HBPAP’s Chairman and the Cabinet Secretary for Health. These leaders ensured that HBPAP’s priority-setting process adhered to the principles outlined in the Internal Procedures Manual such as transparency and participation.

*‘The Cabinet Secretary would always tell us, “You must bring the people along in the process”’* HBPAP participant 7

### Outcome conditions

#### Adequate understanding of the priority-setting process by HBPAP members but limited understanding by non-HBPAP members

HBPAP participants reported that participating in the priority-setting process for health benefits package development had deepened their understanding of the meaning of and need for priority-setting as well as how to conduct priority-setting explicitly.

*‘It oriented me to the importance of explicit priority-setting which offers a clear path to follow as opposed to the usual ad hoc planning where you want to put everything in one basket’.* HBPAP participant 5

Some non-HBPAP participants reported learning substantially by participating in HBPAP’s priority-setting process and equated the learning to a crash course in healthcare financing. However, others reported that they did not understand the process or how it was conducted. They attributed this to their lack of technical knowledge or professional experience in priority-setting and the 60-day timeline that was too short to enable deep sensitization and education of non-HBPAP members.

*‘In the meetings, people were getting confused and surprised. The panel tried to explain their work, but the limitation was time’* MOH participant 3

#### Adequate stakeholder acceptance and satisfaction with HBPAP’s roles, composition and process, but poor acceptance and satisfaction with the final decision-making stage

Concerning HBPAP’s roles, responsibilities and composition, participants reported that majority of HBPAP and non-HBPAP members were satisfied with these. These members felt that HBPAP consisted of experts with impeccable credentials who could competently develop a health benefits package for Kenya.

*‘External stakeholders were happy with the fact that there was a panel. They looked at the mix of people who were there and their professional accolades. They knew this was a team that would do them good’.* HBPAP participant 3

However, participants reported that a few stakeholders such as some MOH bureaucrats and parastatals rejected and were dissatisfied with HBPAP’s roles and responsibilities because they felt that their roles had been usurped and/or their experience in designing health benefits packages overlooked. This lack of acceptance limited HBPAP’s access to official government documents and knowledge of the decisions being made at the senior political level.

*‘The team based at the Ministry has this understanding that nothing can succeed without them. Some felt that this is something they could do, and they did not need anybody from elsewhere to come and do it. Externally, there were stakeholders who felt they should have been the ones to lead the process’* HBPAP participant 7

Concerning HBPAP’s priority-setting process and proposed health benefits package, participants reported that many HBPAP and non-HBPAP members accepted and were satisfied with these. Participants felt that despite time and other resource constraints, HBPAP and non-HBPAP members were adequately involved in a transparent and auditable process that had led to the creation of a scientifically sound benefits package that met the needs of the people.

*‘Everybody that interacted with the panel was very happy with this process and what came out of it’* HBPAP participant 2

However, some HBPAP and non-HBPAP participants criticized HBPAP’s priority-setting process for being overly academic or technical. This undermined stakeholder understanding and acceptance of the priority-setting process. It also undermined incorporation of political views when setting priorities.

*‘One of the panel’s biggest deficiencies was lack of consideration of the political aspect of the process. This was more of a political than a technical process. The panel should have factored in what was politically feasible because this was not an academic exercise but an exercise to inform actual policy.*’ Development partner, participant 1

Concerning the final decision-making process by senior political leadership, HBPAP and non-HBPAP participants expressed their dissatisfaction and poor acceptance due to lack of transparency and consideration of scientific considerations. Participants were also dissatisfied with the lack of publication of HBPAP’s report that detailed the proposed health benefits package given the time, expertise and financial resources that went into developing it. Finally, participants were dissatisfied with the lack of implementation of HBPAP’s proposed health benefits package as this undermined service coverage towards UHC.

*‘Let me explain the disillusionment. The Panel’s creation was a once in a lifetime moment. The panel had a window of opportunity to change how things were done in the health system. However, that window was squandered. Everyone could have done better to support the Panel. How long will it take to recreate that window? When will this ever happen again?’* Development partner, participant 3

#### Limited impact of HBPAP’s priority-setting process on health policy and practice

The outputs of HBPAP’s priority-setting process were a proposed health benefits package and policy recommendations to support health system improvement towards UHC, including proposals for the institutionalization of evidence-based priority-setting processes (Ministry of Health, 2018b). However, the proposed health benefits package was not implemented in the UHC pilot due to several reasons.

First, the senior political leadership changed the UHC model from a health insurance model to a user-fee removal model. This change was thought to be due to concerns about the affordability of HBPAP’s proposed health benefits package. The change to a user-fee removal model was, however, incompatible with the specification of health services and the design of payment methods such as capitation and case-based payments of the proposed health benefits package. In the user-fee removal model, no user fees would be charged in public hospitals in the four pilot counties. Instead, the public hospitals would be reimbursed for services offered using line-item budgets which outline costs of health system inputs as opposed to explicitly defined health services.

*‘The pilot design changed to user-fee removal. The NHIF was no longer responsible for purchasing the UHC benefit package which made it difficult to operationalize the benefit package. The package and its provider payments were designed for a purchaser like NHIF’.*HBPAP participant 1

Second, participants reported that stakeholders’ interests and opposition prevented implementation of the proposed health benefits package. For example, NHIF opposed HBPAP’s new health benefits package because it had its own benefits packages. The NHIF felt that HBPAP had usurped its role for benefits package design. However, senior MOH policymakers felt that the NHIF benefits packages were unaffordable and unsustainable. Private healthcare providers opposed HBPAP’s new health benefits package because it would mean revenue loss from loss of contracts under the government’s proposed implementation plan. Some development partners supporting health financing functions in Kenya opposed HBPAP’s proposed benefits package because they held different opinions on the type of benefits package that should be implemented. Finally, some MOH bureaucrats and NHIF officials opposed HBPAP’s priority-setting process because they felt that they had not been adequately involved.

*‘What happened to the Panel’s benefit package was policy capture. The benefit package was not implemented because of interests from different stakeholders which overshadowed the process.’* HBPAP participant 8

Despite the lack of implementation of HBPAP’s proposed health benefits package in the UHC pilot, HBPAP’s proposed recommendations for health system improvement had influenced several policy reforms. For example, the MOH combined HBPAP’s proposed health benefits package with elements of other existing benefits packages such as the NHIF’s benefits packages and the MOH’s Kenya Essential Package for Health to develop a harmonized benefits package for nationwide implementation towards UHC.

*‘There has been some impact of the panel’s report. The ministry used the report to develop a Harmonized Benefits Package which is a blend of the package proposed by the panel and other existing packages such as Supa Cover, Linda Mama, Civil Servants among others’* MOH participant 1

In response to HBPAP’s recommendation to institutionalize explicit priority-setting through health technology assessment (HTA), the MOH has appointed a focal point for HTA and developed a framework for institutionalizing HTA in Kenya. The MOH has also established a Medicines Affordability Pricing Advisory Committee to use HTA to inform pricing of pharmaceutical products and a HTA Technical Working Group to support development of a strategy for HTA.

*‘One of the annexes in our report is the draft framework for institutionalizing health technology assessment in Kenya. It was a step for the MOH to put in place a systematic process for developing a benefit package because our life was temporary’* HBPAP participant 4

## Discussion

This case study qualitatively evaluated the extent to which HBPAP’s priority-setting process for health benefits package development fulfilled the normative procedural and outcome conditions of a good priority-setting process as set out by Barasa et al. (Barasa *et al.*, 2015). The findings indicate that HBPAP’s priority-setting process partially fulfilled these conditions. This case study offers the following lessons.

While the Barasa et al.’s framework recognizes the interconnection between procedural and outcome conditions, this study further shows the presence of interconnections between specific elements within the procedural and outcome conditions. These interconnections mean that the fulfilment of one element is likely to influence fulfilment of the other. For example, stakeholder participation in HBPAP’s priority-setting process (procedural condition) influenced the extent of transparency and incorporation of community values (procedural conditions) as well as stakeholder understanding, acceptance and satisfaction (outcome conditions). Similar findings have been reported in macro level priority-setting processes in Uganda (Kapiriri and Be Larose, 2019), UK, New Zealand, Australia and Canada (Mitton *et al.*, 2006) and meso level priority-setting processes in Kenya (Bukachi *et al.*, 2014; Waithaka *et al.*, 2018b) and Zambia (Tuba *et al.*, 2010). Lack of transparency in the final decision-making stage in HBPAP’s priority-setting process (procedural condition) undermined stakeholder acceptance and satisfaction (outcome condition). Comparable findings have been made in macro level priority-setting processes in Korea (Ahn *et al.*, 2012). Finally, limited stakeholder acceptance and satisfaction (outcome conditions) with HBPAP’s roles and proposed health benefits package undermined the impact of HBPAP’s process on policy and practice (outcome condition).

This study also showed that partial fulfilment of the procedural and outcome conditions could be attributed to factors internal to the priority-setting process such as HBPAP’s multidisciplinary and multi-stakeholder composition, HBPAP members’ technical expertise, finances and time allocated for the process and availability of internal procedures manual. These findings are supported by international literature. For example, the multi-stakeholder composition of priority-setting bodies influenced stakeholder participation and inclusiveness in macro level priority-setting processes in Australia (Whitty and Littlejohns, 2015). The technical expertise of the members of priority-setting bodies influenced their extent of participation and empowerment as well as the extent of external stakeholders’ acceptance and satisfaction with macro level priority-setting processes in Australia, UK, New Zealand and Canada (Mitton *et al.*, 2006). Limited allocation of financial resources influenced external stakeholder involvement in meso level priority-setting processes in Kenya (Nyandieka *et al.*, 2015; Waithaka *et al.*, 2018b), Zambia (Zulu *et al.*, 2014) and Tanzania (Maluka *et al.*, 2010a; 2010b) and macro level priority-setting processes in Australia (Whitty and Littlejohns, 2015). Limited allocation of time for the priority-setting process influenced stakeholder understanding in Canada (Gibson *et al.*, 2006) and use of evidence and stakeholder participation in Kenya (Nyandieka *et al.*, 2015; Waithaka *et al.*, 2018b) and Tanzania (Maluka *et al.*, 2010b). Finally, the availability of manuals and guidelines influenced enforcement and degree of transparency and evidential requirements in macro level priority-setting processes in Australia (Whitty and Littlejohns, 2015) and UK (Mitton *et al.*, 2006) and meso level processes in Kenya (Barasa *et al.*, 2017).

Our study further showed that partial fulfilment of the procedural and outcome conditions could be attributed to factors external to the priority-setting process, such as legal instruments (gazette notice, the constitution and national policy documents), quality of evidence for priority-setting criteria and external stakeholders’ technical expertise or experience in priority-setting. These findings are supported by international literature. For example, legal instruments influenced legitimacy, transparency, stakeholder participation, use of evidence and availability of appeals, revisions and enforcement mechanisms in macro level priority-setting processes in Australia (Whitty and Littlejohns, 2015), Germany (Kieslich and Littlejohns, 2012) and Chile (Charvel *et al.*, 2018), as well as meso level priority-setting processes across countries of different income levels (Waithaka *et al.*, 2018a). Limited quality of evidence undermined use of criteria in macro level priority-setting processes in the UK, Australia and Canada (Mitton *et al.*, 2006) and meso level priority-setting processes in Kenya (Waithaka *et al.*, 2018b) and Tanzania (Maluka *et al.*, 2010a; 2010b). External stakeholders’ technical expertise and experience in priority-setting influenced their level of participation in priority-setting processes in Kenya (Wanjau *et al.*, 2021), UK (Robinson *et al.*, 2012) and Tanzania (Maluka *et al.*, 2010a).

This study also highlighted the political nature of priority-setting processes and the undermining influence of politics on a priority-setting process. Despite HBPAP establishing an explicit priority-setting process, political interference and stakeholder interests undermined transparency of the decision-making process, use of evidence, stakeholder acceptance and satisfaction with the roles and process and impact of HBPAP’s recommendations on health policy and practice. Priority-setting processes are inherently political given conflicting opinions on what procedures and evidence should be followed, who should be involved and what roles they should play (Ham and Glenn, 2003). Existing literature shows that clarity and acceptance of roles influenced stakeholder participation at the meso level in Kenya (Barasa *et al.*, 2017). Political interference and donor interests influenced final decision-making in macro level priority-setting processes in Kenya (Wanjau *et al.*, 2021), Uganda (Kapiriri *et al.*, 2007) and meso level priority-setting processes in Kenya (Bukachi *et al.*, 2014; Waithaka *et al.*, 2018b), Tanzania (Maluka *et al.*, 2010b) and Zambia (Tuba *et al.*, 2010). Similarly, private sector interests (e.g. pharmaceutical industries) have also influenced final decision-making in macro level priority-setting processes in Australia (Mitton *et al.*, 2006) and Korea (Ahn *et al.*, 2012).

Given these findings, several strategies may be put in place to strengthen the priority-setting process for health benefits package development in Kenya. Publicity and transparency of the process can be increased through publication of reports using contextually appropriate modes of communication. Allocation of adequate time and financial resources can facilitate wider stakeholder involvement, identification of stakeholder preferences and application of criteria. Improvement of health information systems and generation of local empirical studies and contextualized thresholds can improve quality and use of criteria. Stakeholder training and continuous involvement in priority-setting processes can empower them and increase their understanding and acceptance of the process. Finally, managing politics and stakeholder interests can enhance the success of priority-setting processes. This includes, for instance, mapping stakeholders and their interests and actively engaging them to obtain negotiated buy-in for the process. It also includes establishing clear procedural and decision-making frameworks that explicitly demarcate stakeholders’ roles, responsibilities and powers to minimize political interference in decision-making.

Our study shows that the Barasa et al.’s framework offered a simple yet adequate approach for not only describing but also evaluating HBPAP’s priority-setting process, considering the normative conditions of a good healthcare priority-setting process. While the framework recognizes the interconnection between procedural and outcome conditions, our study further highlights the presence of interconnections between specific elements within procedural and outcome conditions. In addition, by exploring why procedural and outcome conditions were partially met, our study identified internal and external factors that influenced the extent to which HBPAP met these normative conditions. Future researchers seeking to apply the Barasa et al.’s framework to evaluate healthcare priority-setting processes should not only explore interconnections within and across procedural and outcome conditions but also explore internal and external factors that might influence the extent to which the priority-setting body fulfils these conditions.

## Study limitations

This case study involved a retrospective account of a process conducted over 2 years ago which may have led to recall bias. However, this was mitigated through document reviews which are effective in retrieving accounts of past events (Bowen, 2009). The study respondents may have provided answers that they perceived as desirable leading to social desirability bias, but this was mitigated through triangulation of data using document reviews (Bergen and Labonté, 2020). With snowball sampling, it is possible that HBPAP members selected participants with similar views, but this was mitigated through document reviews. While not all stakeholders involved in HBPAP’s priority-setting process were interviewed, document reviews make it unlikely that conducting more interviews would have led to greater depth in the findings. Finally, participation by some of the authors in previous priority-setting processes in Kenya may have biased the interviews and analysis, but this was mitigated through document reviews and peer debriefing sessions.

## Conclusion

This case study describes and qualitatively evaluates HBPAP’s priority-setting process for health benefits package development, thus contributing to existing literature on evaluation of macro level priority-setting processes in LMICs. It demonstrates the value of evaluating existing priority-setting processes against the key conditions of an ideal priority-setting process as outlined in empirically and theoretically informed evaluative frameworks. It also demonstrates the interconnectedness of the elements within and across the procedural and outcome conditions. While a priority-setting process may be structured to be explicit and systematic, its procedural and outcome conditions may be partially fulfilled due to internal and external factors. Areas of partial fulfilment provide possible opportunities for strengthening the process. Importantly, priority-setting processes are inherently political; thus, managing politics and stakeholder interests is key in enhancing the success of priority-setting processes.

## Supplementary Material

czac099_Supp

## Data Availability

The data underlying this article cannot be shared publicly due to ethical reasons. As such, we wish to maintain the anonymity and confidentiality of all participants involved in the study. The data will only be shared on reasonable request to the corresponding author.

## Reflexivity

The authors include four females and one male and span multiple levels of seniority from PhD fellow to associate and full professors. Two of the authors are health economists while three are health systems experts. All authors have extensive experience in health systems research in examining health financing arrangements and health policies in LMICs in sub-Sahara Africa and Asia.
