# Supplementary material for: A qualitative evaluation of priority-setting by the Health Benefits Package Advisory Panel in Kenya
Source: Health Policy Plan. 2022 Nov 14;38(1):49–60. doi: 10.1093/heapol/czac099 (PMC9849713; doi:10.1093/heapol/czac099)
Supplement: czac099_Supp [file czac099_supp.zip › Supplementary material.docx]

Supplementary material: Definition of the procedural and outcome conditions outlined in the evaluation framework

| **Procedural condition** | **Definition** |
| --- | --- |
| Transparency and Publicity | Information on the priority-setting process including procedures followed, evidence considered, stakeholders involved, decisions made and rationales for the decisions made is openly and publicly available. |
| Use of evidence | The reasons (such as economic, scientific, or social considerations) upon which the priority-setting and resource allocation decisions are made. |
| Stakeholder participation and incorporation of community values | Relevant health system stakeholders attend meetings, provide their inputs (shared beliefs, values, and principles) and contribute to the process of setting priorities and making decisions on the allocations of resources. |
| Empowerment | The extent to which power differences among stakeholders are minimized and opportunities for stakeholder participation maximized to enable contribution to and ability to influence decisions. |
| Appeals and revisions mechanism | A mechanism through which the priority-setting process and resource allocation decisions can be challenged, reconsidered, and amended given emerging evidence or concerns over the validity of the process or evidence used. |
| Enforcement | A mechanism for ensuring that all the other procedural conditions are adhered to. |
| **Outcome condition** | **Definition** |
| Stakeholder understanding | Stakeholders gain knowledge of the goals, structure, rationales, and outcomes of the priority-setting process. |
| Stakeholder acceptance and satisfaction | The extent to which stakeholders regard the priority-setting process and decisions as valid (adequate or suitable) and the extent to which they are willing to support and participate in future priority-setting processes. |
| Impact on health policy and practice | Extent to which changes in priorities or distribution of resources as well as changes in health system policies can be credibly linked to the priority-setting process. |
